# HD-DRUM, a Tablet-Based Drumming Training App Intervention for People With Huntington Disease: App Development Study

**DOI:** 10.2196/48395

**Published:** 2023-10-06

**Authors:** Claudia Metzler-Baddeley, Monica Busse, Cheney Drew, Philip Pallmann, Jaime Cantera, Vasileios Ioakeimidis, Anne Rosser

**Affiliations:** 1 Cardiff University Brain Research Imaging Centre (CUBRIC) School of Psychology Cardiff University Cardiff United Kingdom; 2 Centre for Trials Research School of Medicine Cardiff University Cardiff United Kingdom; 3 Jimi Can Bristol United Kingdom; 4 Department of Psychological Medicine and Clinical Neurosciences School of Medicine Cardiff University Cardiff United Kingdom; 5 Cardiff Brain Repair Group School of Biosciences Cardiff University Cardiff United Kingdom

**Keywords:** Huntington disease, electronic health, intervention, training application, rhythm, timing, drumming, movement, cognition, integrated knowledge translation, gamification, Template for Intervention Description and Replication (TIDieR), TIDieR, mobile phone

## Abstract

**Background:**

Huntington disease (HD) is a neurodegenerative condition that leads to progressive loss of cognitive-executive and motor functions, largely due to basal ganglia (BG) atrophy. Currently, there are no therapeutic interventions tailored to address executive and motor dysfunction in people with HD. Music-based interventions may aid executive abilities by compensating for impaired BG-reliant timing and rhythm generation using external rhythmic beats. Here, we applied an integrated knowledge translation (IKT) framework to co-design a tablet-based rhythmic drumming training app (HD-DRUM) to stimulate executive and motor abilities in people with HD.

**Objective:**

The primary aim was to develop the HD-DRUM app for at-home use that addressed the accessibility needs of people with HD and allowed for the quantification of performance improvements and adherence for controlled clinical evaluation.

**Methods:**

The IKT framework was applied to iteratively refine the design of HD-DRUM. This process involved 3 phases of knowledge user engagement and co-design: a web-based survey of people with HD (n=29) to inform about their accessibility needs, usability testing of tablet-based touch screens as hardware solutions, and usability testing of the design and build of HD-DRUM to meet the identified accessibility needs of people affected by HD and their clinicians (n=12).

**Results:**

The survey identified accessibility problems due to cognitive and motor control impairments such as difficulties in finding and navigating through information and using PC keyboards and mouses to interact with apps. Tablet-based touch screens were identified as feasible and accessible solutions for app delivery. Key elements to ensure that the app design and build met the needs of people with HD were identified and implemented. These included the facilitation of intuitive navigation through the app using large and visually distinctive buttons; the use of audio and visual cues as training guides; and gamification, positive feedback, and drumming to background music as a means to increase motivation and engagement. The co-design development process resulted in the proof-of-concept HD-DRUM app that is described here according to the Template for Intervention Description and Replication checklist. HD-DRUM can be used at home, allowing the quantification of performance improvements and adherence for clinical evaluation, matching of training difficulty to users’ performance levels using gamification, and future scale-up to reach a wide range of interested users.

**Conclusions:**

Applying an IKT-based co-design framework involving knowledge user engagement allowed for the iterative refinement of the design and build of the tablet-based HD-DRUM app intervention, with the aim of stimulating BG-reliant cognitive and motor functions. Mapping the intervention against the Template for Intervention Description and Replication framework to describe complex interventions allowed for the detailed description of the HD-DRUM intervention and identification of areas that required refinement before finalizing the intervention protocol.

## Introduction

### Background and Rationale

Huntington disease (HD) is an inherited progressive neurodegenerative disease where cell loss in basal ganglia (BG) networks of the brain manifests as cognitive decline, loss of motor control, and mood disturbances. Striatal atrophy [1] and white matter degeneration [2] are observed many years before the onset of movement symptoms. These early brain changes are accompanied by impairments in psychomotor speed and executive functions [3,4] including problems in decision-making, multitasking, and motor sequence learning, all of which may hamper a person’s everyday functional abilities such as working capacity [5].

Currently, there are no disease-modifying treatments for HD, and only a very few studies on HD-specific cognition-oriented interventions have been conducted [6]. However, accumulating evidence suggests that neurologic music therapy (NMT) in the form of rhythmic auditory stimulation (RAS) and therapeutic instrumental music performance training [7] may be beneficial in the rehabilitation of acquired brain injuries [8-11] and neurodegenerative diseases, including HD [12] and Parkinson disease (PD), which is another movement disorder that affects the BG [13-15]. Recent reviews and meta-analyses have concluded that music-based interventions may be beneficial for gait, timing of upper extremity functions, and quality of life after stroke [8] and may improve gait and mobility in people with PD [13] and motor and cognitive functions in people with HD [12]. NMT interventions such as RAS are thought to compensate for the loss of BG-generated timing and rhythm signals with external rhythmic cueing [16]. Other potentially related mechanisms include accelerated learning due to increased temporal skills through rhythmic movement practices and motivational aspects of musical rhythm [17]. However, the clinical effects of NMT interventions and the mechanistic underpinnings in the brains of people with HD remain unknown because evidence from high-quality randomized controlled trials is still scarce.

Previously, in 2 pilot studies, we explored rhythmic movement training (bongo drumming) as a therapeutic tool for people with HD [18,19]. We chose drumming because it is an activity that requires the learning, planning, and execution of movement sequences, which are all abilities that depend on BG function and become more difficult as the disease progresses. Thus, we hypothesized that drumming would not only improve motor abilities and response speed but also cognitive functions involved in the planning and execution of movements and multitasking.

In our pilot research [18,19], people with HD followed audio instructions that taught them to drum along on a pair of bongo drums. Each 10- to 15-minute training session introduced a novel drumming pattern, starting with simple, slow patterns and gradually increasing in complexity and speed. We found that 2 months of drumming training at home was acceptable for people at the premanifest and early stages of the disease and was associated with significant improvements in cognition and white matter microstructure. Anecdotally, some patients and their spouses reported the benefits of hand coordination and general alertness. However, the training delivery on bongo drums also had some disadvantages. First, it did not allow for the quantification of training-induced performance improvements or the recording of adherence, that is, the duration and frequency of training engagement. Second, as drumming responses were not tracked, the level of training difficulty could not be matched to an individual’s performance level to avoid over- and underchallenge. Third, delivering the training on CDs and bongo drums made an increased scale of use more challenging compared with the wide accessibility of digital apps on tablets and smartphones.

An electronic health app for drumming training that can be delivered on smartphones or tablets has the potential to address these issues. However, little is known about the use of digital technologies and any potential barriers to use in people with HD, with the exception of research on wearable or portable sensors to monitor motor and cognitive alterations in people with HD [20,21]. A recent review of the literature not only concluded that digital technologies hold promises for therapeutic research and symptom management in people with HD but also added that the devices need to be standardized and protocols harmonized to optimize their clinical use in people with HD [20].

Electronic health apps are increasingly being used as cost-effective and widely accessible tools to support health care delivery, monitoring, and education [22,23]. With the recent explosion of the electronic health market, ensuring that electronic health apps are appropriately designed to meet the needs of end users before introducing them as health interventions has become increasingly important. Similarly, it has been recognized that published descriptions of electronic health interventions often lack sufficient detail to replicate or evaluate their effects.

Recently, the UK Medical Research Council and National Institute for Health Research updated their framework for the development and evaluation of complex interventions including eHealth [24]. The new framework recommends engaging stakeholders, addressing uncertainties, refining intervention and theory, and considering the context of intervention delivery and economy as core elements to guide research into complex interventions [25]. These recommendations reflect the growing support within health research for co-design, that is, the engagement of knowledge users in the development and evaluation of interventions [26]. Knowledge users refer to any stakeholders involved in influencing, administering, or using the health care system, including those with lived experiences. One framework that has been developed to facilitate co-design is the integrated knowledge translation (IKT) approach that refers to an interactive process of knowledge exchange between different stakeholders to produce interventions that are useful to health care system knowledge users [27]. Within the context of eHealth, the US Institute of Medicine has identified usability (the extent to which an end user can use a product to achieve specified goals) as a key component of good practice for the development of electronic apps [28]. Therefore, the usability of an electronic health app needs to be tested by knowledge users as part of the developmental process to ensure that end users’ needs are met. Thus, the engagement of knowledge users in co-design and the iterative usability testing for design refinement have emerged as important components of the IKT approach for the development of electronic health interventions [29].

We adopted the IKT framework to develop a tablet-based rhythmic movement (drumming) training app intervention for people with HD. This paper first reports the IKT-based intervention development process and then provides a detailed description of the intervention using the Template for Intervention Description and Replication checklist [30]. This paper focuses on the documentation of the data that were acquired during the iterative co-design development process including data from background research into the use of digital technology in people with HD and from usability testing of hardware and software options. The proof-of-concept HD-DRUM app described here is suitable for clinical evaluation.

### Objectives

The primary aim of this research was to develop HD-DRUM, a tablet-based drumming training app intervention for people with HD.

## Methods

### Overview

We developed a proof-of-concept tablet-based drumming training app called HD-DRUM for clinical evaluation. The purpose of the HD-DRUM intervention was to provide a digital training platform that provided solutions to the challenges encountered with the use of the bongo drumming training that we previously investigated. Specifically, it allowed for the (1) quantification of performance improvements, (2) quantification of training engagement, (3) matching of the training difficulty to performance levels, and (4) scale-up to reach for a wider audience via delivery on tablets or smartphones.

**Figure 1:**
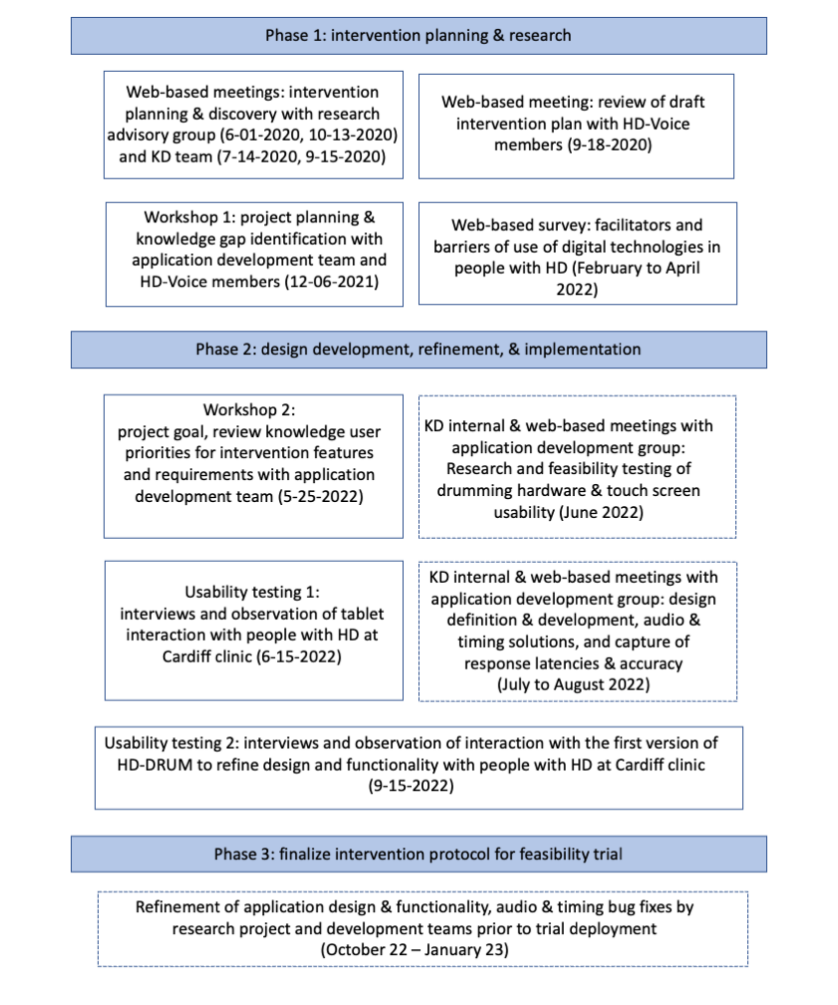
Overview of the integrated knowledge translation approach of the intervention development process. HD: Huntington disease; KD: Kinneir Dufort.

Following the IKT framework and in collaboration with the user-centered design and innovation agency Kinneir Dufort (KD) [31] and knowledge users, we applied a dynamic and iterative model of development that included knowledge user engagement at different stages of the planning and development of HD-DRUM (Figure 1).

### Ethical Considerations

This project has received favorable ethical opinion from the Wales Research Ethics Committee 2 (reference: 22/WA/0147) and the School of Psychology Research Ethics Committee at Cardiff University (EC.21.12.14.6493). The project was sponsored by Cardiff University (SPON1895-22). All participants provided informed consent. The privacy and confidentiality of participants were protected in accordance with the Data Protection Act of 2018. All study data were coded and deidentified.

### Participants and Recruitment

For this study, the following 4 groups were formed:

1. The app development group comprised the lead researcher (CM-B), the KD design and engineering team, and musician JC. This was the core team that designed and built the HD-DRUM app in collaboration with the knowledge users and the research advisory group.
2. The research advisory and management group comprised an interdisciplinary team of researchers, experts, and clinicians working in fields related to the project and research area. This group consisted of 5 researchers (CM-B, AR, CD, MB, and PP) with expertise in psychology, neuroscience, intervention development and evaluation, clinical trial methodology and statistics, and neurology. This group was established before starting the IKT development process to help plan the intervention development, approach, research questions, and outcome measures and to monitor the progress of the project in line with milestones, on a monthly basis.
3. The knowledge user group comprised people affected by HD, including those with HD, their carers, family members, and clinical staff working in the HD field. A total of 5 people affected by HD were members of HD-Voice, the patient and public involvement group of the UK Huntington’s Disease Association, a third-sector support organization. Furthermore, 29 individuals with HD were recruited via an advertisement in the Huntington’s Disease Association newsletter, and they participated in a web-based survey on the facilitators of and barriers to the use of digital technologies. Demographic information on age, gender, education, and disease stage for these participants was collected as part of the web-based survey. In addition, knowledge users were recruited for usability assessments from the Cardiff University HD clinic, and 7 people participated in the usability assessment of tablet interactions in people with HD. These were 4 people with early to moderate manifest HD, 2 carers, and 1 clinician. Another 4 people with HD at various stages of disease progression (premanifest to manifest) and 1 member of the clinical staff participated in usability testing of the first version of the HD-DRUM app.
4. The research project team comprised 3 core research members responsible for running the feasibility trial into
  HD-DRUM. This group consisted of the lead researcher (CM-B), the postdoctoral research associate (VI), and Doctor of Philosophy students and was established during the finalization phase of the app development. Members of this group piloted and tested the app before the trial deployment.

### Intervention Development

#### Phase 1: Intervention Planning, Research, and Feasibility

##### Overview

Members of the research advisory group met via a web-based conference tool and communicated via email to review an updated project plan based on the findings of the previous pilot studies [18,19]. They discussed the research questions, the research approach, and outcome measures of the updated project in preparation for a funding application. In parallel, CM-B engaged with the KD team to develop a road map for the design of the new intervention app that incorporated knowledge user engagement and iterative usability testing throughout the development process. The project plan was reviewed in a web-based meeting between the lead researcher and members of HD-Voice, who provided feedback on the feasibility of the planned intervention and research protocol for people with HD. To minimize participant burden and increase retention rates, it was suggested to shorten the originally proposed training duration of 6 months to 2 months and to limit assessments before and after the intervention and at corresponding time points for the control group in the feasibility trial. It was also suggested to develop a stand-alone app version for offline use to include individuals without an internet connection in the research. This feedback was incorporated into the research protocol.

Once funding was secured for the project, the app development team met in a hybrid workshop with HD-Voice members to review the project plan and identify uncertainties and knowledge gaps that needed clarification before designing an app solution. Specifically, the need to gain an understanding of how people with HD use digital technologies, what barriers they encounter, and what design and functionality requirements need to be met when designing and building the app were identified. Given the lack of evidence in the literature, we conducted a web-based survey of the facilitators of and barriers to the use of digital technologies by people with HD.

##### Web-Based Survey Into the Use of Digital Technologies in People With HD

The survey consisted of 21 items and was delivered and analyzed using Qualtrics (version 2022; **Qualtrics** International Inc) [32]. Items included (1) the frequency of use of different digital devices (mobile phones or smartphones, tablet, and PC or laptop) and the internet; (2) the preferred digital device; (3) the type of digital activities carried out (emails; social networking; buying goods or services; listening to music; downloading information, games, and images; and video calls); and (4) how confident participants felt in conducting these activities as rated on a 5-point scale ranging from “not at all” to “very confident.” Furthermore, participants were asked to provide information as to whether they required any assistive technologies or accessibility settings and whether they had encountered any problems when using digital devices.

#### Phase 2: Design Development, Refinement, and Implementation

At the start of this phase, the development team and a member of the research advisory group met to align all team members on the project goal, review, and build knowledge including a discussion of the web-based survey results. In addition, the prioritization of various app components was discussed. The KD team then researched different hardware and software configurations suitable for a drumming app including drumming on a tablet touch screen and drumming on connected or standard bongos. Direct drumming on touch screens had the advantage of reducing costs, complexity, and technical risks (from additional circuitry and choice of audio transducers) but required usability testing with people with HD.

To assess the feasibility of tablet interactions in people with HD, we engaged with the service users of the Cardiff-based HD clinic. The freely available bongo drumming app *Congas & Bongos* [33] was installed on 2 different tablets of varying screen sizes: a 10-inch Samsung Galaxy (Samsung Electronics) and a 12.9-inch iPad Pro (Apple Inc). For comparability purposes, the “4- and 5-finger swipe multitasking gesture” on the iPad Pro was deactivated before the test. The participants were approached in the clinic waiting room and encouraged to interact with the bongo drumming app on both tablets. The tablets were placed on a table placed in front of the participants. The participants were asked the screen size that they preferred. The research team made notes on any feedback and suggestions from the participants and any observations on how people interacted with the app.

The app development group then started the design process, which involved close collaboration between the KD team, JC who recorded the audio and timing content, and CM-B who monitored the alignment with project goals and specified requirements. Core elements of the development process concerned the app design, implementation of its audio and timing features as well as response latency and accuracy capture. During the design definition and development phase, different design solutions were iteratively tested and refined internally by KD to ensure accessibility, functionality, usability, and delight. Different solutions for audio and timing specifications and latency capture were investigated and evaluated in terms of functionality, technical risk, and cost-effectiveness.

The first version of the HD-DRUM app was then tested for its usability in people with HD in the Cardiff HD clinic. Usability testing was conducted in the waiting area of the Cardiff HD clinic to refine the app design to the needs of people with HD by observing how participants interacted with the app and by gaining feedback and suggestions about what worked well and what needed to be improved. The tablet was placed in front of the participants on a table sufficiently close to them, so that they could reach the tablet comfortably, and the participants were encouraged to interact with the app. Researchers observed these interactions involving opening and navigating through the app and engaging with the first training sessions, made notes, and collated feedback.

#### Phase 3: Finalization of the Intervention Protocol for the Feasibility Trial

Design and functionality refinements based on the usability findings were implemented. The app was iteratively tested and corrected for any remaining bugs including audio and timing errors by the project research and development teams. The result was the HD-DRUM app intervention outlined and described in Textbox 1. Textbox 2 provides an overview of the data recorded and their intended objectives.

**Description of the HD-DRUM app (version 1.0) intervention according to the Template for Intervention Description and Replication framework.**

**Name and description of intervention**

- HD-DRUM, a tablet-based drumming training app intervention aimed at stimulating cognitive and motor abilities in people with Huntington disease (HD).

**Why: rationale, theory, and goal or goals**

- Rationale: HD causes neurodegeneration in the basal ganglia, leading to a progressive loss of cognitive and motor control. Drumming involves the learning of rhythmic motor sequences, and our pilot research suggested benefits of bongo drumming on executive functions and white matter microstructure in the corpus callosum and in pathways between the right supplementary motor area and putamen in people with HD [18,19]. The holistic evaluation of the potential therapeutic benefits of drumming in HD requires a digital training solution that allows (1) the quantification of performance improvements and training engagement, (2) the matching of the training difficulty to performance levels to avoid over- or underchallenge, and (3) scale-up to reach a wider audience via delivery on tablets or smartphones. The HD-DRUM app was designed to address these points and to meet the accessibility needs of people with HD.

**What: materials and procedures**

**Figure 2:**
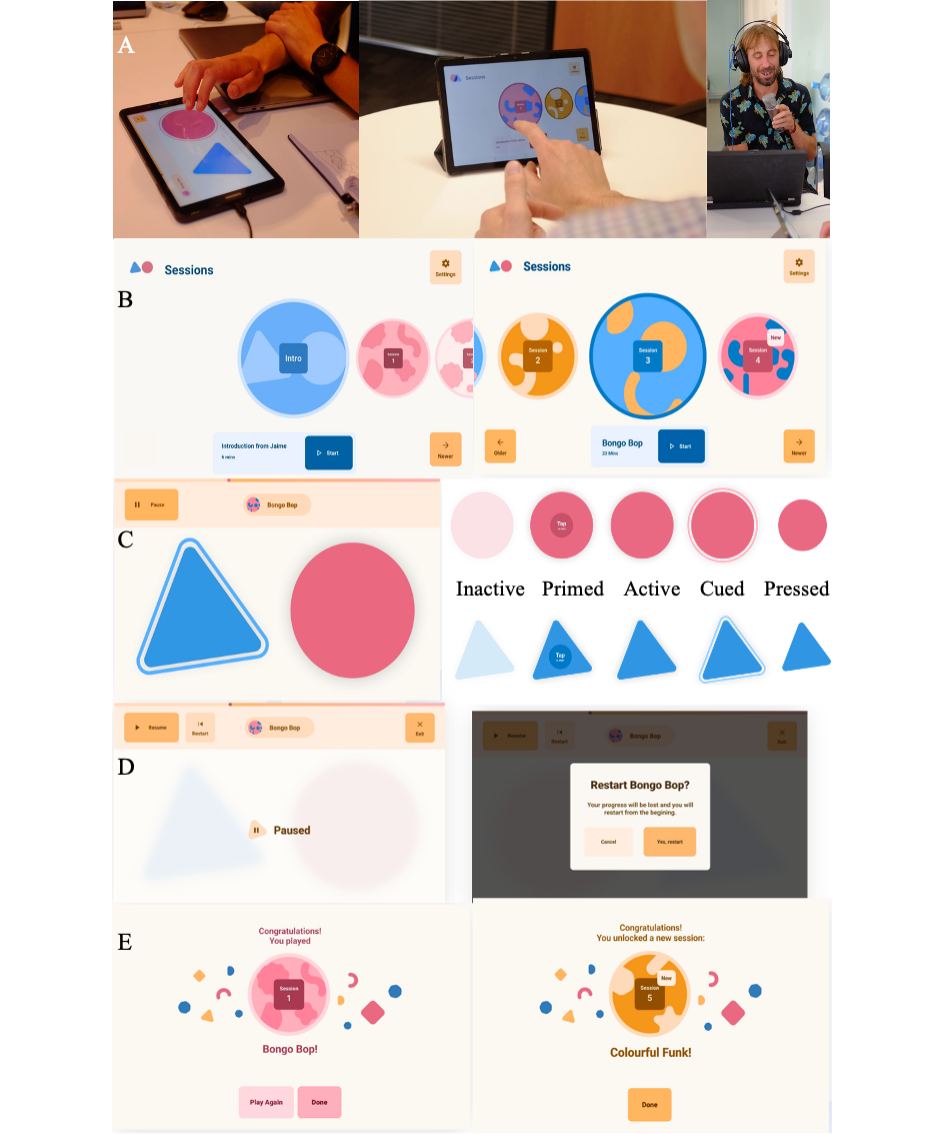
An overview of the HD-DRUM app design. (A) The HD-DRUM app comprises 22 audio training sessions that were recorded by musician JC and runs on a Samsung Galaxy 8 tablet. (B) The home screen displays the introduction and all the unlocked training sessions. Users can scroll through and start the sessions. (C) The drum screen displays 2 web-based drums, a blue triangle and a red circle, that respond with high- and low-pitch bongo sounds when tapped. A visual halo cues the expected hit during the performance sessions. (D) Training can be paused at any time. A confirmation screen appears when participants select restart or exit the session before completing it. (E) At the end of each session, the user receives positive feedback congratulating them on completing the session or for having unlocked the next session.

- The HD-DRUM app has been programmed in native Android Java and runs on the Android operating system (version 21 or higher). The chosen hardware was a Samsung Galaxy Tablet A8 with a 10.5-inch screen (Figure 2A). A demonstration of the app is provided here (Multimedia Appendix 1). Participants were provided with the app on a tablet in a protective case and with a 17-page instruction manual.
- Participants start the app by tapping on the HD-DRUM icon on the tablet home screen. HD-DRUM consists of 23 audio recordings, including 1 introduction and 22 training sessions, with each spanning 10 to 15 min. Each training session introduces rhythmic patterns that are based on paradiddles (patterns where a single tap is followed by a double tap, for example, right tap, left tap, right tap, right tap, left tap, right tap, left tap, left tap), and different rhythmic styles, including Hip-Hop, Funk, Samba, and Reggaeton (a form of dance music that originated in Puerto Rico and fuses Latin rhythms, dancehall, and hip-hop or rap). Patterns are practiced with and without a metronome or background music. The shape and color of each session’s identity are unique and reflect the session’s rhythm and background track (Figure 2B). The first 3 sessions introduce slow and regular rhythms and are available to all users right from the beginning. Through the program, the patterns then gradually increase in tempo and complexity with more target beats to hit, longer beat sequences, and more complex rhythms.
- After starting the app, the introduction, the first 3 sessions, and any unlocked sessions are displayed on the HD-DRUM home screen, and participants can scroll through them (Figure 2B). A session is active and can be started when its circle icon is displayed enlarged in the center of the screen. A session can be started by either tapping on the session icon or by pressing the start button. When a drum session is opened, 2 drums, a blue triangle and red circle, appear in transparent colors on the screen and need to be tapped to start the audio instructions (Figure 2C). Alternatively, the start button can be pressed. Participants can pause the session at any time. When the session has been paused, there are 3 options: resume, restart, or exit the session (Figure 2D). A bar on the top of the screen shows the participants how far they have progressed through the session.
- In each training session, participants are instructed to tap or drum along as per the audio instructions on the 2 drums (the blue triangle and the red circle) on the tablet screen. They are told to tap with the tips of all fingers simultaneously. The drums mimic physical Bongo drums by producing visual (shrinking) and audio feedback (a high-pitch bongo sound for the left triangle and a low-pitch sound for the right circle) when tapped.
- In sessions 1 to 7, the rhythmic patterns are first practiced with each hand separately and then with both hands together; after session 8, all patterns involve both hands and are practiced starting with one hand and then in reverse order with the other.
- Each session consists of training and performance parts. During the training, participants learn and practice to tap along to a new rhythmic pattern. During performance parts, participants are asked to tap or drum along as accurately, that is, as synchronized as possible with the recorded bongo sounds, while their response accuracy and latencies are recorded. To facilitate accurate performance while responses are recorded, drumming is guided by a visual halo cue that appears around the target drum when a hit is expected during performance parts only (Figure 2C).
- The interaction with each training session produces an output file in comma-separated format that is stored on the tablet and uploaded to a project-specific space on Google Firebase (Google LLC) [34] when the tablet is connected to the internet. The output files contain a record of the latencies and accuracies of all tap responses and their mean statistics to quantify performance improvements and a time stamp including the duration of the training engagement to quantify adherence (see Textbox 2).
- HD-DRUM data collection complies with General Data Protection Regulation and data privacy regulations. No personal or identifiable user information are transmitted or stored on the tablet or on Google Firebase. HD-DRUM output files are coded with a 16-digit-letter-string tablet ID. Firebase services encrypt data at rest and during transit using http secure.
- Each session audio file is accompanied by a Musical Instrument Digital Interface (MIDI) file that contains information about the timing of the audio drumming sounds. Accuracy levels are determined by comparing the time stamps of the sounds in the MIDI file with the recordings of participants’ responses to the audio instructions. An accurate response in HD-DRUM is defined as a correct left- or right-hand tap within a predefined, symmetrical time window around the expected hit stored in the MIDI file. On the basis of the previous findings of simple and choice reaction times in HD [35] and our own usability testing in the Cardiff clinic, the largest time window for the slowest rhythmic pattern (<1 beat/s) in session 1 was set to 1000 ms, that is, 500 ms before and after the expected hit. Time windows were then gradually reduced to 100 ms (50 ms before and after expected hit) for rhythmic patterns with tempos over 100 beats/min (maximum 117 beats/min).

**Who provided**

- The Cardiff University research team provided participants with the app for home training.

**How delivered**

- Participants engage with the training sessions on an individual basis at home. If possible, family members and carers will be engaged to facilitate the training by providing a supportive environment.

**Where delivered or required infrastructure**

- The stand-alone tablet-based training is delivered at participants’ homes. Participants are instructed to place the tablet flat on a table in front of them at a comfortable reaching distance. Internet connection is not required for training delivery.

**When and how much**

- Participants are instructed to practice for approximately 10-15 min/d, 5 times/wk, for 8 wk.

**Tailoring**

- The app includes an element of gamification such that from session 3 onward, participants must reach an accuracy level of ≥70% to unlock the next more difficult session. If their performance accuracy fell below this threshold, they were encouraged to repeat the session or practice one of the previous sessions. At the end of each session, participants received positive feedback for having either completed the session (for accuracy levels of <70%) or unlocked the next session (for accuracy levels of ≥70%; Figure 2E).

**Modifications**

- The current version of HD-DRUM is a proof-of-concept prototype. The feasibility evaluation will inform future modifications to the app.

**How well: planned**

- Adherence with the training is remotely monitored by the research team via the output files that are generated during the app engagement and uploaded onto Google Firebase when the tablet is connected to the internet. The research team monitors adherence and progression remotely and stays in weekly contact with participants via email, text, or phone calls.

**How well: actual**

- The feasibility assessments are ongoing.

**Mapping of the data measured in HD-DRUM and their intended objectives.**

**Quantification of performance improvements**

- Low-level tap events (accurate responses are defined as correct left- or right-hand taps within a predefined time window around the time stamp of the expected hit from the Musical Instrument Digital Interface file. Accurate responses are recorded as “1” and incorrect responses, missed hits, or additional taps within a time window are recorded as “0” in the output file)
  Left tap response latencies (ms)
  Left tap response accuracies
  Right tap response latencies (ms)
  Right tap response accuracies
- Summary statistics
  Mean tap response latency (ms)
  Mean tap response accuracy
  Mean left tap response latency (ms)
  Mean right tap response latency (ms)
  Minimum (best) left tap response latency (ms)
  Minimum (best) right tap response latency (ms)
  Mean left tap response accuracy
  Mean right tap response accuracy

**Quantification of adherence**

- Date and time of training engagement
- Duration of training engagement

**Metadata to quantify training session difficulty**

- Total number of target beats
- Duration of allowed time window (ms) for hits
- Success accuracy threshold (%)

## Results

### Digital Technologies Survey

#### Participant Demographics

A total of 15 female and 14 male individuals aged >25 years (19/29, 66% aged between 35 and 65 y) completed the survey anonymously (Multimedia Appendix 2). Half of these individuals were university educated at the undergraduate (6/29, 21%) or postgraduate (9/29, 31%) level, including 2 (N=29, 7%) who had a doctoral degree. Of the remaining participants, 6 (N=29, 21%) had an A-level or bachelor of technology, 4 (N=29, 14%) had a general certificate of secondary education, and 4 (N=29, 14%) had other educational or vocational qualifications. Most participants reported being at the premanifest (8/29, 28%) or early disease stage (17/29, 59%), whereas 4 (N=29, 14%) reported middle or later stages.

#### Use of Digital Technologies, Facilitators, and Barriers

Multimedia Appendix 2 presents the main results of the web-based survey. Most participants (25/29, 86%) reported using the internet daily (24/29, 83% multiple times), and 7% (2/29) used it once a week or once a month (Multimedia Appendix 2).

The most popular devices were smartphones (16/29, 55%) and tablets (9/29, 31%; Multimedia Appendix 2), with most participants using them daily (25/29, 86% for smartphones and 10/29, 34% for tablets). PCs and laptops were used by 52% (15/29) at least once a day, but only 10% (3/29) preferred them over other devices.

Participants who used digital devices and the internet engaged in a range of activities including sending or receiving emails; social networking; purchasing goods and services; reading news; downloading games, images, films, and news articles; playing music; and participating in video calls (Multimedia Appendix 2). Overall, the majority felt fairly confident or very confident in carrying out these activities, ranging from 62% (18/29) for downloading news and social networking to 79% (23/29) for listening to web-based music (Multimedia Appendix 2).

However, some participants reported that they never used a device (15/29, 52% for tablets, 6/29, 21% for PC or laptops, and 3/29, 10% for smartphones) and did not feel confident in carrying out the activities mentioned in the previous paragraph (ranging from 1/29, 3% for listening to web-based music to 3/29, 10% for participating in video calls and social networking). In addition, 7% (2/29) responded that they had never used the internet. However, given that they responded to an internet-based web-based questionnaire, they perhaps received assistance from carers, family members, or friends in participating in the survey.

Although most individuals (24/29, 83%) reported that they did not require any assistive technology or accessibility settings, 2 participants mentioned that they used password reminders and 1 participant had the spouse help with web-based banking. One participant (1/29, 3%) did not know which assistive technology was available.

The problems that participants experienced when using digital technologies fell broadly into those related to cognitive deficits, primarily attention and executive deficits, and motor impairments (Multimedia Appendix 3). For instance, participants reported difficulties in understanding instructions, having to enlarge pages and taking a long time to find or not being able to find information or apps on their phone. Motor problems included difficulties with dexterity and grip when operating digital devices including problems with typing on a small keyboard, using a PC mouse, taking a long time to type, and accidentally clicking on incorrect information. These difficulties seemed to become apparent with advancing age and the development of clinical symptoms, as all participants who reported barriers were at the manifest disease stages.

#### Summary and Conclusion of Survey Results

In summary, the responses to our survey suggested that most participants engaged with digital technologies and used smartphones, tablets, and PC or laptops for numerous web-based activities. However, the responses to our questions about problems with using apps identified some accessibility issues related to cognitive and motor problems, notably difficulties in understanding instructions and finding information, as well as difficulties with dexterity and grip that affected typing on a keyboard and using a mouse. These accessibility issues would need to be addressed in the app design for people with manifest HD.

### Usability Testing of Tablet Interactions in People With HD

We tested our assumption that interactions with tablet touch screens of 2 different sizes were feasible for people with HD at the Cardiff clinic.

#### Screen Size and Position

We found that both the large and small tablets performed well in terms of the participants being able to tap on the bongo drums on the screen. Participants did not indicate a preference for one screen size. However, when asked, some participants preferred the larger device owing to differences in screen and sound quality. Multiple participants said they owned a tablet, but not all were engaging with it. One participant who participated in the original bongo drumming pilot study said that they preferred the tablet to the physical bongos. More important than the screen size was the close placement of tablets for people to reach. Multiple participants moved the tablets closer to them because reaching too far out led to many faulty presses.

#### Interaction With the App

The participants used their index finger or index, middle, and ring fingers together to tap the tablet. One participant with manifest HD alternated between 1 and multiple fingers because of the limited ability to control the movement. For some people with manifest HD, initiating the first tap appeared to require considerable effort and concentration; however, the subsequent interactions appeared easier. Some impulsive and perseverative responses were observed, that is, the same tapping interaction was repeated with minimal control of speed or force. The audio feedback upon tapping on the tablet-based bongos was positively received. The app showed drums of 2 different sizes, but it was agreed that this was not particularly necessary for our project. Multiple participants appeared joyful while playing, and their body language demonstrated comfort when immersed in the drumming interaction.

#### Motivation and Engagement

Multiple participants mentioned the need to gamify the app to maintain engagement. Some participants played PC games and stated that the app would have to be more engaging than games such as Solitaire. Multiple participants also mentioned the need to have music alongside drumming to keep the training interesting and engaging. The use of headphones that could be plugged into the app was received well, as it helped the participants focus more and would prevent family members from listening in or being bothered by the noise. A family member mentioned the potential mental health benefits of engaging in the drum-playing activity. Multiple participants thought it would be helpful to be reminded to drum on the app as a method of engagement. The 15-minute sessions were generally accepted as a feasible length, but the participants were hesitant to suggest that this was a comfortable length of time. A participant discussed with their carer or carers that it would be fun to be able to play together, perhaps alongside other instruments.

#### Outcome of Tablet Usability Testing

Successful drumming on the screen of both tablet sizes suggested that a tablet-based version of the app using bongos on the screen would be feasible for use by people with HD. The smaller 10-inch Samsung Galaxy was chosen as a more cost-effective and accessible hardware solution than the iPad Pro. It was evident that the provision of clear instructions regarding the tablet position on the table and the hand gesture used for tapping on the tablet-based drums (playing with the tips of index, middle, and ring fingers) to facilitate interaction with the app was important. User testing indicated that the addition of reinforced screen protection and bumper cases for improved durability was necessary to mitigate the difficulties in controlling the force and speed of tapping. Furthermore, an element of gamification and positive feedback should be included in the app design, as well as the option to play to background music to keep the training engaging.

### Usability Testing of the HD-DRUM App

Usability testing of the first version of the HD-DRUM app was conducted to gain feedback for refining the interface to ensure accessibility, functionality, usability, and delight.

#### Interaction With the App

A 10-inch Samsung Galaxy tablet with the app installed was placed on a table in front of the participants at a comfortable distance for them to tap on the tablet surface. Participants were asked to start the app from the tablet home screen by tapping on the HD-DRUM icon and navigate through the app by scrolling through the training sessions and starting, pausing, and exiting them.

Participants were then encouraged to engage in the first training session. Each training session involved listening to audio instructions by the musician (JC) and tapping or drumming as per his instructions on 2 drums on the tablet screen (Figure 2). When tapped, the left drum, shaped as a blue triangle, produced a high-pitch bongo sound and the right drum, a red circle, a low-pitch bongo sound. Participants were first instructed to distinguish between these sounds and associate them with the left and right drums. They were then asked to synchronize their drumming movements as accurately as possible with the audio beats played by JC. To illustrate this, JC demonstrated audio examples of “accurate” (synchronized) and “not so accurate” (desynchronized) playing along. To help with the learning of the beats, JC provided additional left and right verbal cues, as illustrated in the following transcribed example of the instruction for the first training session:

Let’s get started. With the left hand we are going to tap in all beats 1, 2, 3, and 4. Nice and slow. So only with your left hand, tap along with me on your left bongo, the triangle. Come on, let’s start. [Audio left high-pitch bongo drumming starts and JC counts the beats]: 1, 2, 3, 4; 1, 2, 3, 4; 1, 2, 3, 4; 1, 2, 3, 4; left, left, left, left; left, left, left, left, 1, 2, 3, 4; 1, 2, 3, 4;1, 2, 3, 4; 1, 2, 3, 4...

for about 1 min

Very good, now we are going to do the same thing, but I will not be calling out the numbers or say left or right. You just tap along with me on your left bongo. Ready? Let’s go for it!

Audio left high-pitch bongo drumming starts and plays for about 1 min

Ok, now we will do the same thing but on the right hand. So first I will help you calling out the numbers and then you do it on your own. So now you will tap with your right hand only on your right circle bongo. Let’s go for it. [Audio right low-pitch bongo drumming starts and JC instructs]: right, right, right, right; and right, right, right, right; and 1, 2, 3, 4; 1, 2, 3, 4; 1, 2, 3, 4; 1, 2, 3, 4; right, right, right, right...

for about 1 min

Very good. Now without the voice just with the sound of the bongo with your right hand.

Audio right low-pitch bongo drumming starts and plays for about 1 min

Ok, now we are going to drum with both our hands. Left and right and left and right. To help you with the timing you will hear the sound of a high-hat such as this one [demonstrates high-hat sound] in between each beat. Let’s go. [Audio left high-pitch, high-hat, right low-pitch bongo drumming starts]: Left, right, left, right, left, right.

1 min

Now we are doing this again for a bit longer without so much of my talking. Let’s go for it!

Audio left high-pitch, high-hat, right low-pitch bongo drumming starts and plays for 1 min

Each training session consisted of a learning phase, such as the aforementioned example, and a performance phase during which the participants’ performance accuracy was measured. Accurate responses were defined as the responses on the target bongo drum within a prespecified time window around the target beat. Guided by the literature on finger tapping and simple and choice reaction time latencies in people with HD [35,36], the first training session involved slow and regular target beats, with a timing window of 1000 milliseconds. Over the course of the 22 training sessions, the duration of these timing windows was gradually reduced as the tempo of the target beats increased (Textbox 1).

All participants were able to use and navigate through the app. One participant with manifest HD required one-to-one instruction initially but then appeared to progress afterward. The participants enjoyed the voice-over by the musician (JC). Specifically, they found his instructions engaging and clear and enjoyed the intonation of his voice, which was of an English speaker with a native Spanish accent, as illustrated by the following quote:

I actually liked the voice. Easy to follow. Very clear...I don’t take auditory info very well, and for me it was ok to understand.

Participants also enjoyed the look and feel of the interface, which was called “fresh, clean, and clear.” Overall, participants appeared happy and joyful when interacting with the app.

All the participants understood the task of drumming along as per the audio instructions. Participants also seemed to understand the different expectations between the training and performance parts of the intervention and were able to complete the performance sessions. A visual halo cue alongside auditory feedback seemed to help further identify the beat on which to drum, as specifically expressed by some participants or simply observed by us in others. The addition of background music provided a good challenge for some participants, one of whom noted that it made them concentrate more. A training session length of approximately 10 to 15 minutes was considered acceptable. However, longer training sessions were not acceptable.

Participants commented that they enjoyed the element of gamification by having to reach a certain success level to unlock the next training session and the positive feedback at the end of the session that congratulated them for having completed the session.

#### Refinements to the App Content and Instructions

Participants reported that the introduction session that explained how to use the app was too long and detailed, with some confusion about whether they were supposed to drum along during the introduction. Therefore, the introduction session was shortened to make it more focused and concise.

Some people with manifest HD found the first training rhythm of 65 beats per minute too fast for an introduction session. They tended to tap on all 4 beats instead of the single beat as instructed. This seemed to have arisen due to the confusion and difficulty in distinguishing between the spoken metronome and target beat. In response, we included a slower session of >1 beat per second with regular tapping on all 4 beats without the metronome as the introductory session.

Participants predominantly used single fingers over flat hands and needed explicit instructions to tap on the tablet-based drums with a flat hand using all fingers together to mimic bongo drumming. To compensate for this, the app instruction manual included detailed instructions on how to tap correctly. Participants received face-to-face instructions and demonstrations by the research team at the start as part of the intervention delivery.

#### Refinements to the App Design

##### Home or Session Screen

When participants interacted with the session icons on the screen (Figure 2B), they expected that tapping on a full-size, centered icon would start the session and that tapping on a minimized icon would scroll and center that session instead of using the arrow and play buttons at the bottom of the screen. Therefore, these functionalities were added to the app design. The introduction session was not recognized as having different content compared with the training session and was therefore made visually distinctive with an introduction label. The session icon artwork was grouped into threes, with regard to colors and shapes to reflect the background track, which was the same for 3 sessions in a row.

##### Pause Screen

The pause screen caused confusion, and there was a risk of participants accidentally pressing “exit” or “restart” instead of “resume” and losing their progress as a result. Consequently, the design was changed to a more prominent (in size and location) *resume* button to reflect the fact that *resume* was the main action, and *restart* and *exit* were secondary action choices. Furthermore, a confirmation step after pressing “restart” or “exit” was added to avoid users making these choices by mistake.

##### Drum and End-of-Session Screens

Static icons were replaced with dynamic icons to reflect the session that was playing and to allow the user to finish the session by either tapping the icon of the session just played, if this was to be repeated, or by tapping the icon of the newly unlocked session.

#### Summary

Overall, the participants were positive about the app design and the training content. They were able to navigate the app and follow the audio instructions. They appreciated the visual halo cues and auditory feedback when tapping the tablet-based drum, as well as playing along with background music and the gamification element of the app. However, some refinements to the design and functionality of the screen displays, the length and content of the introduction, and the first training session, in addition to more detailed instructions regarding the tapping responses, were required to maximize accessibility, functionality, usability, and enjoyment of the app design.

## Discussion

### Principal Findings

HD leads to a progressive decline in the control of executive and motor functions, largely due to atrophy in BG networks, with detrimental effects on a person’s ability to function in everyday life [5]. Currently, there are no therapeutic interventions tailored to address executive and motor dysfunction in people with HD. Music-based therapies have been reported to benefit cognitive and motor functions in people with PD [13-15] and HD [12,18,19]. It has been proposed that strong rhythmic beats may compensate for impaired BG-reliant timing and rhythm generation by providing external cues for the planning and execution of movements [16]. Music may also facilitate learning through rhythmic practices and motivational aspects of musical rhythm [17]. However, the clinical effects of music-based interventions in people with HD and their neural mechanisms remain unknown due to a lack of evidence from high-quality clinical trials.

The objective of this research was to develop a tablet-based drumming training app, HD-DRUM, that uses rhythmic beats to stimulate BG-mediated cognitive and motor functions. HD-DRUM was developed to enable the objective evaluation of performance improvements and adherence in clinical trials and to address the accessibility needs of people with HD in an engaging way.

For this purpose, we adopted an IKT-based co-design approach that involved knowledge user engagement in focus groups and iterative usability testing. The IKT process enabled an interdisciplinary research team to engage with a design company, a musician, and knowledge users in a semiformalized way to formulate, co-design, and refine the intervention app. By involving people with HD in the app development process, we were able to identify and address accessibility barriers and refine the design to maximize the accessibility, functionality, usability, and enjoyment of interactions with the interface. The co-design highlighted the importance of designing the app in a user-friendly and intuitive manner, with a focus on key functionalities to avoid cognitive overload.

We started the research process by identifying knowledge gaps in the literature regarding digital technology use and its barriers among people with HD. Thus, we conducted a web-based survey to identify the accessibility needs that would need to be addressed in the design of the HD-DRUM app. Subsequent iterative usability testing informed the feasible and accessible hardware and software design solutions for people with HD.

The survey identified a number of accessibility issues associated with attention or executive and motor control impairments in people with HD, such as difficulties in finding information, navigating through apps, as well as lack of dexterity and fine motor control to type on keyboards and use PC mouses to interact with apps. These difficulties were reported by individuals with manifest HD; hence, they appeared to become more apparent with disease progression and the associated loss of executive functions and motor control in people with HD. Executive dysfunction may manifest as problems with focusing attention, distractor suppression, task-switching, planning, and problem-solving [4,37,38] that are known to interfere with everyday activities [39]. The reported problems also conform to the loss of fine motor abilities and the control of upper limb and hand movements in people with HD [40]. To the best of our knowledge, this is the first report of how these clinical symptoms may affect the use of digital technologies in people with HD.

On the basis of these findings, we decided to implement the HD-DRUM app on tablets with touch screens that did not require typing on a keyboard or using a mouse. Behavioral observations of participants’ interactions with the touch screens and apps and their feedback from the usability testing were instrumental in the implementation of key features of the app. Usability testing confirmed that a touch screen on a 10-inch tablet was a feasible and accessible solution for people with HD from the premanifest to manifest disease stages. The tablet solution allowed for the implementation of drums on the touch screen to capture the participants’ responses (Figure 2). This solution addressed the difficulties due to motor symptoms by facilitating easy navigation through the app by tapping on large and visually distinctive buttons (Figure 2). Accessibility issues due to attention problems and cognitive overload were addressed by keeping the design clean and simple, focusing only on key functionalities (scrolling through sessions, starting, pausing, resuming, and exiting), and by having clear and concise audio instructions supported by a combination of audio and visual feedback (bongo sounds and shrinking drums); verbal instructions (left, right, and counting); and visual halo cue training aids (Figure 2). Multimodal learning cues were implemented based on evidence that cueing may help achieve better movement performance by compensating for attentional deficits [41,42].

Furthermore, elements of gamification by rewarding participants for reaching a learning target with unlocking the next training session and positive feedback at the end of each session were implemented to maintain motivation and engagement [43]. To keep the training varied and interesting, different rhythmic styles were chosen as training materials and practiced with and without background tracks or metronome beats (Textbox 1). Participants enjoyed these aspects of the training and thought that they would help with training adherence. These features that aim to maintain motivation and engagement may be of particular importance for people with HD who are affected by mood disturbances, apathy, and a lack of motivation.

The co-design development process resulted in the proof-of-concept HD-DRUM app that is described here according to Template for Intervention Description and Replication (Textbox 1) to provide details of the intervention’s rationale, key elements, design, and functionality for future replication. To the best of our knowledge, the HD-DRUM is the first tablet-based rhythmic training app developed with and for people with HD.

HD-DRUM addresses the methodological shortcomings of our previous pilot research on the feasibility of a bongo drumming intervention in people with HD [18,19].

The implementation of the bongo drumming intervention as a digital tablet-based app allowed for the quantification of performance improvements by recording the latencies and accuracies of participants’ tapping responses on the tablet-based drums. Furthermore, adherence to the training was quantified by recording the frequency and duration of participants’ engagement with the app. This allows for the objective assessment of the feasibility of using HD-DRUM at home.

On the basis of knowledge user feedback, participants’ performance levels were matched to the training difficulty by means of gamification, such that a success criterion of 70% accuracy [44] needed to be reached before the next training session could be unlocked. Matching users’ practice to a level appropriate to their abilities is expected to increase the acceptability of the intervention by avoiding frustration and boredom due to over- and underchallenge.

Furthermore, the tablet-based app format of the training that can be used for home practice has the potential to widen accessibility and increase the scale of use and hence will allow the scale-up of sample sizes in future randomized controlled trials.

These features of the HD-DRUM app allow for the objective assessment of the feasibility and clinical effects of the training in a controlled clinical trial. This will enable researchers to address the lack of evidence from high-quality randomized controlled trials on the clinical effects and efficacy of this type of NMT.

HD-DRUM is currently deployed in a randomized controlled trial to assess the feasibility of 2 months of at-home HD-DRUM intervention compared with usual activity control in people with HD. Adopting the IKT framework enabled us to design the HD-DRUM app in a way that maximizes the possibility of trial success by considering the needs of people with HD during the development process.

### Limitations

It is important to note some limitations of the co-design development process adopted here. Only a small group of people with HD, albeit at different disease stages, participated in the usability testing. Their feedback may not be representative and may not address the accessibility needs of all people with HD. Similarly, conducting a web-based survey about the use of digital devices and accessibility barriers would have been biased toward those individuals who were able to use the internet in the first place. The percentage of the population with HD that we did not reach with this type of survey is unknown to us. A more inclusive way of reaching a representative sample of people with HD may be by asking participants to fill in the survey at their clinic visits or posting it to their homes.

With regard to the developed proof-of-concept HD-DRUM app, tapping on drums on the screen may not provide the full sensory experience of drumming on real bongo drums and may perhaps be less effective than playing on a real instrument. Similarly, individual training at home may be less effective than engaging in a drumming circle because it may lack the benefits of social contact and the experience of producing music together. By contrast, the app delivery may make the training more accessible to individuals who are not mobile and cannot attend a social music gathering. The challenge lies in balancing the need for an intervention that allows for the evaluation of performance improvements under controlled conditions against capturing the multiple mechanisms that may underlie the benefits of music-based interventions.

### Conclusions

The findings of the feasibility study may help address some of these questions and may shape future modifications and refinements of HD-DRUM. In the future, HD-DRUM may be able to provide a remotely accessible training tool to help maintain or improve movement and cognition in people with HD without the risk of harmful side effects. In the future, it may be feasible to combine novel disease-modifying therapies with behavioral interventions such as HD-DRUM to maximize therapeutic outcomes for people with HD. Even a small delay in the onset of symptoms would have direct and significant benefits for the quality of life of people with HD and their families.

## Appendix

Multimedia Appendix 1 Video example of a participant engaging with a training session of the HD-DRUM app. The participant has learned the rhythm well and is anticipating the tapping response accurately.

Multimedia Appendix 2 Main findings of the web-based digital technology survey of 29 individuals with Huntington disease. (A) Use of different devices; (B) preferred device, activities for which the devices were used, and requirements for accessibility settings; and (C) confidence ratings for individual activities.

Multimedia Appendix 3 Barriers to using digital technologies reported by survey participants.

## Abbreviations

BG: basal ganglia
HD: Huntington disease
IKT: integrated knowledge translation
KD: Kinneir Dufort
NMT: neurologic music therapy
PD: Parkinson disease
RAS: rhythmic auditory stimulation

## Data Availability

The data sets generated and analyzed during this study are not publicly available to protect participants’ privacy and confidentiality due to the small number of participants and the rarity of Huntington disease but are available from the corresponding author upon reasonable request.
