# Supplementary figures and images for: HD-DRUM, a Tablet-Based Drumming Training App Intervention for People With Huntington Disease: App Development Study
Source: JMIR Form Res. 2023 Oct 6;7:e48395. doi: 10.2196/48395 (PMC10589837; doi:10.2196/48395)

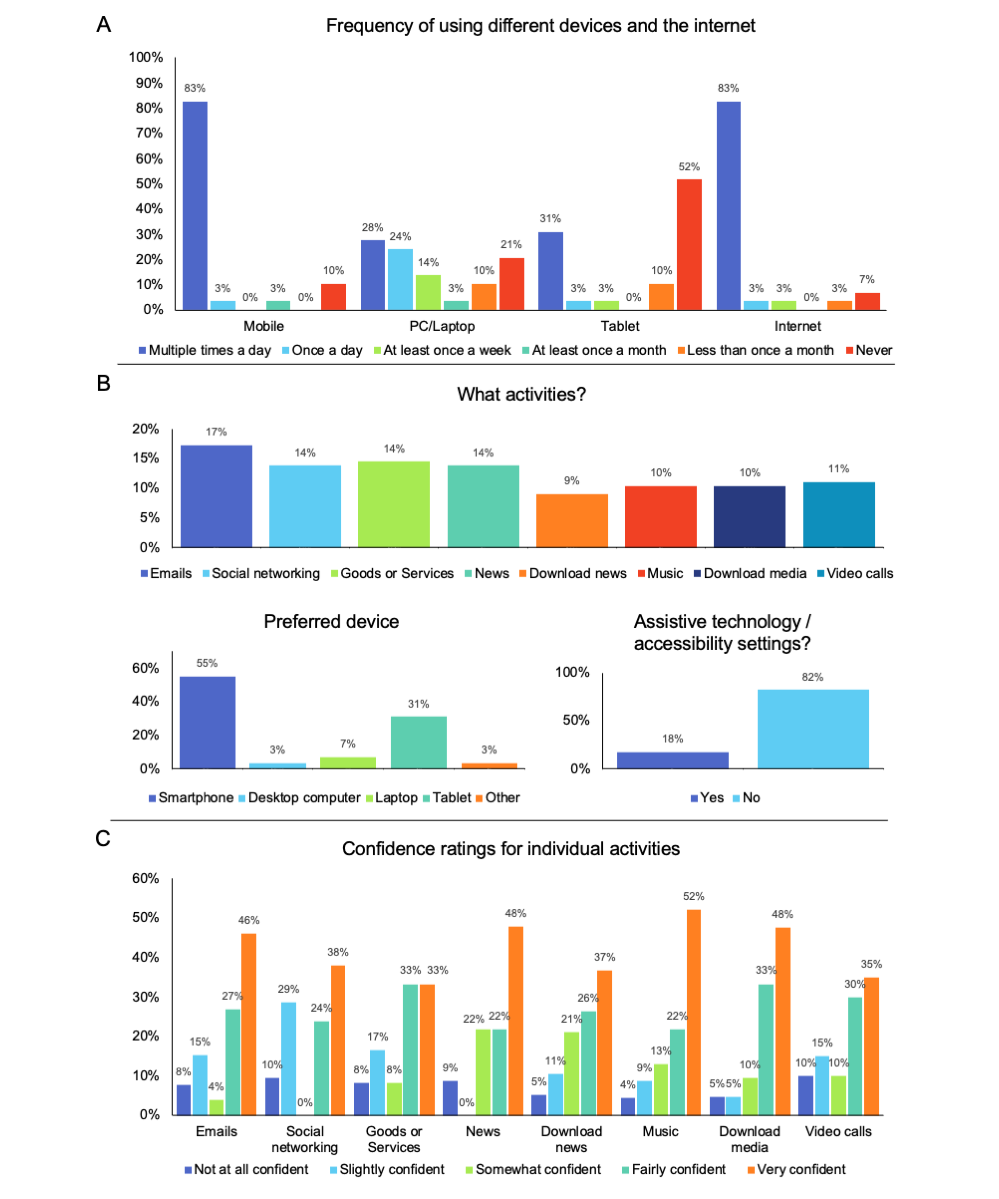

Supplement: Multimedia Appendix 2 [file formative_v7i1e48395_app2.png]
