# Supplementary material for: HD-DRUM, a Tablet-Based Drumming Training App Intervention for People With Huntington Disease: App Development Study
Source: JMIR Form Res. 2023 Oct 6;7:e48395. doi: 10.2196/48395 (PMC10589837; doi:10.2196/48395)
Supplement: Multimedia Appendix 3 [file formative_v7i1e48395_app3.docx]

Supplementary Table: Barriers to using digital technologies reported by survey participants

| **HD stage** | **Assistive technology/ accessibility settings** | **Problems** |
| --- | --- | --- |
| **early** |  |  |
|  | Password reminder | I can find it difficult to understand what is being asked at times or can’t find information. I can take a long time to find applications that I use on my iPhone frequently. |
|  | - | I struggle with a mouse on the computer so bought a tablet instead |
|  | - | Yes, accidentally clicking on the incorrect letters and words when using automatic texting. I don’t use voice activated smart devices like Siri as already fed up repeating myself with people |
|  | - | I have to enlarge the page and it takes me so long to type, it’s very frustrating |
|  | - | I have to type really slow, often people don't think I’m going to answer |
|  | - | Problems with online banking - my wife helps me |
|  | - | Find it hard typing on small keyboard |
| **later** |  |  |
|  | I don’t know what is available. | My lack of dexterity and grip mean that I am unable to operate any electronic/digital devices. |
|  | - | I can read but not send emails. I cannot use applications. |
